# Environmental sensitization, advocacy and resource-management training in a resource-limited paediatric surgical educational setting

**DOI:** 10.3389/fmed.2026.1823289

**Published:** 2026-10-29

**Authors:** Marion Arnold, John Lazarus, Sharon G. Cox

**Affiliations:** 1 Red Cross Children’s War Memorial Children’s Hospital, Division of Paediatric Surgery, Department of Surgery, Faculty of Health Sciences, University of Cape Town, Cape Town, South Africa; 2 Division of Urology, Department of Surgery, Faculty of Health Sciences, University of Cape Town, Cape Town, South Africa

**Keywords:** advocacy, education, environmental sustainability, gastroschisis, low-to-middle income country, paediatric surgery, sustainable development, teratogen

## Abstract

**Aim:**

A policy framework for Paediatric surgical training to address sustainable surgical resource utilization and environmental-disease interplay in a low-to-middle income country (LMIC) setting is presented.

**Methods:**

Using the United Nations Educational, Scientific and Cultural Organization (UNESCO) Education Pillars for Sustainable Development (EPSD), existing and potential integration of environmental education in undergraduate and postgraduate paediatric surgical training is described at an LMIC academic hospital, in conjunction with a narrative review of environmental underpinnings of existing paediatric surgical English literature. Pedagogical techniques are conceptualized to demonstrate the 3-way interactions between paediatric surgical services and social/ built, economic and natural/ climactic environments, on individual and community level. This is illustrated by the context of the surgically managed congenital abdominal wall defect gastroschisis, amongst other examples.

**Results:**

Gastroschisis is a Paediatric Surgical key topic that highlights the imperative for comprehensive environmentally sensitive patient-orientated care. Some curricular content and LMIC situational awareness already exists in our setting regarding resource stewardship, environmental teratogens and socioeconomic interactions. The local surgical procedure-related carbon footprints and interaction with socio-economic factors are inadequately researched to inform resource management and educational content. Topical paediatric surgical literature and local knowledge, attitude, skills and participation have significant gaps on individual, institutional and community levels, aggravated by a circular interplay between these UNESCO EPSD. Potentially feasible Paediatric Surgery curricular changes to increase environmental focus include explicit standard pedagogical content, behaviour modelling/ modulation and interdisciplinary engagement.

**Conclusions:**

Paediatric Surgery curriculum and educational and healthcare culture currently convey little environmentally implicit or explicit pedagogical content, and require teaching of skills for critical thinking, research, resource management and organized advocacy.

## 1 Background

Surgical care contributes significantly to the environmental impact of healthcare, which is responsible for nearly 5% of global greenhouse gas emissions (1). Education is deemed essential to address environmentally sustainable surgical practice (1), and aligns with Target 4.7 of the unanimously adopted 2015 United Nations Sustainable Development Goals (SDG) aimed for by 2030, whereby green principles are embedded into national curricula, as well as SDG 12 and 13, addressing responsible consumption and production as well as urgent climate action (2). Environmental changes also directly and indirectly impact educational curricula, requiring mitigation for effective training (3). In our academic paediatric surgical context, despite a culture of frugality borne of fiscal constraints prevalent in our low-to-middle income country/context (LMIC), sophisticated stapling and energy devices have been increasingly used to improve patient care. These single-use plastic devices, together with consumable wrappings, disposable drapes and gowns and other surgical waste contribute 20 to 70% of total hospital waste (1), while there are carbon emissions from operating theatre heating, lighting, ventilation and air-conditioning and anaesthetic gases drive between a quarter to six times the hospital carbon footprint.

The impact and awareness of interplay between climate changes and paediatric surgical disease is growing although poorly understood. The United Nations Educational, Scientific and Cultural Organization (UNESCO) has called for a new paradigm of lifelong value-based learning to stimulate individual and systemic change to address the collective crises of widening social and economic disparity, climate change, biodiversity loss, unsustainable resource depletion as well as challenges from rapid technological change (3). Unfortunately, formal education on environmental-healthcare interplay is minimal in most paediatric surgical academic settings. Environmental decline requires this paradigm to change, but direct, rapid and effective management of disease burden dominates training schedules. We aimed to assess the local training emphasis on environmental concerns within the academic discipline of Paediatric Surgery, identify scope for improvements, using a core knowledge topic (gastroschisis) as a case study, and create a framework for enhanced environmental sustainability integration within a resource-limited setting.

### 1.1 Assessment of current curricula and framework for shaping change

Our undergraduate curriculum provides paediatric surgery training to 5th year medical students through an 8-week period of combined paediatric and paediatric surgical lectures, small-group tutorials and bedside teaching, as well as comprehensive online resources including an online textbook. Formative and summative assessments include presentations of case studies and multiple-choice questions. Total duration of dedicated paediatric surgical exposure is under 2 weeks.

For qualification as a paediatric surgeon after completion of medical school, internship and obligatory community service in South Africa, a further period of dedicated surgical training is required, of at least 5 years within a university-affiliated in-house institutional programme. In addition, a research mini-thesis is required towards a Master of Medicine degree. This is awarded following presentation of an adequate portfolio of operative cases including ongoing clinical work-based assessments and several examinations culminating in a final theoretical exit examination. The apprentice-style training of surgical specialists under the supervision of qualified surgeons is driven by a practical clinical focus and partly self-directed, with dedicated pedagogical lectures or tutorials for on average 3 h a week in our unit.

This descriptive narrative considers how the United Nations Educational, Scientific and Cultural Organization (UNESCO) Educational Pillars for Sustainable Development (EPSD) of awareness, knowledge, attitude, skills and participation (4) are and can further be applied to improve environmentally sensitive healthcare for training and trainer paediatric surgeons as well as medical undergraduates within our paediatric surgical curriculum. Pedagogical techniques for improving environmentally sound practices must incorporate not only cognitive but also socio-emotional, practical and behavioral components to stimulate the individual to shift institutions and communities towards pro-environmental care (5), and are explored.

Gastroschisis is a striking congenital abdominal wall birth defect for which management is core to paediatric surgical training. Throughout the manuscript, this diagnosis is used to illustrate the interplay of environmental factors on the condition and its management, including possible unmeasured risk factors for adverse outcomes related to the environment on an individual, social, economic and environmental level, and associated educational opportunities. These are summarized in Figure 1.

**Figure 1:**
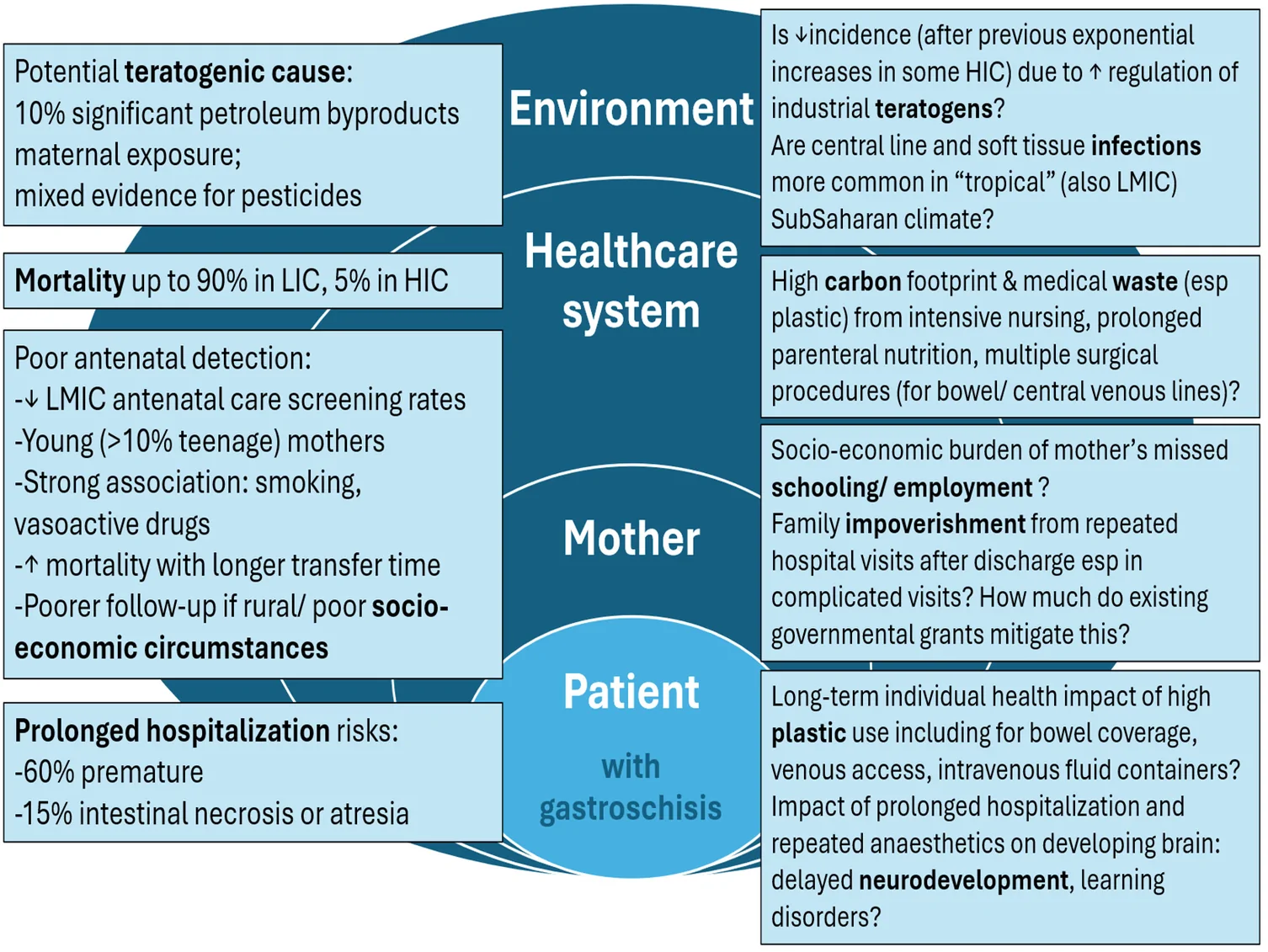
Known (left) and unmeasured (right) factors in interplay between the patient with gastroschisis and the social, built, economic and natural environment, low income countries (LIC); high income countries (HIC), low-to-middle income countries (LMIC).

A narrative literature review of English-language grey literature and peer-reviewed publications on gastroschisis, “green” paediatric surgery, the paediatric surgery “carbon footprint” and paediatric surgery and the environment, using Google Chrome, Microsoft Edge, Google Scholar and Pubmed supplements our exploration of local ecological practices integrated within our healthcare sector to inform paediatric surgical pedagogical content.

Current practices and discussion of future potential paediatric surgical curriculum enhancements are integrated for conciseness.

## 2 Creating awareness

### 2.1 Awareness of the impact of the environment on paediatric surgical conditions

Environmentally explicit paediatric surgical curriculum content at undergraduate and postgraduate (paediatric surgical trainee) level is limited to teaching about teratogenic causes of surgically-treated birth defects (6) within the curricula. In a LMIC, the implicit environmental impact on disease presentation is physically apparent with delayed presentations and poor outcomes associated with adverse socio-economic conditions as well as weather phenomena leading to increased trauma-related pathology (e.g., Winter seasonal peak in flame burns and scalds from informal settlements). Sensitivity to these risks is particularly relevant in our highly unequal country, where over 18% of our city's population live in informal settlements (7).

The unusual epidemiology of gastroschisis, which disproportionately affects teenage mothers (8), could be used to stimulate further discussion about possible environmental teratogenic variables. Clusters of presentation can provoke conversations around teratogen exposure: “they come in three's” (a stochastic effect seen in rare conditions). The rarity of the disease, limited and poor clinical evidence base for causative teratogens, social marginalization of young mothers as well as poor antenatal detection rates in a low-to-middle income country (LMIC) context mean this discussion is limited in textbooks on the topic, despite 22 causative teratogens for gastroschisis identified in animal studies (9) and 10% of mothers of babies with gastroschisis having significant petroleum byproduct exposure in a human study (10, 11). These same limitations have prevented adequate graduate level research on the topic necessary to support advocacy efforts to reduce the incidence of the distressing and costly condition.

South African gastroschisis incidence (0.07 to 2.1 per 10 000 live births (12–14) remains lower than the 3 to 4 per 10 000 live births reported in the United Kingdom (UK) (15) and United States of America (USA) (16). Previous dramatic rises in the incidence of gastroschisis in high income countries (HIC) such as the UK and USA over the past 2 decades are now declining (15, 17, 18), compared to ongoing steady rising but overall lower incidence in South Africa and other African countries (19). Global paediatric surgical research initiatives have compared clinical outcomes between HIC and low income countries (LIC) (20), but research is needed to understand why the incidence trends between HIC and LMIC differ. Are these dissimilar incidence trends due to poor tracking of birth defect rates, genetic predisposition or differences in teratogen exposure? Use of textbooks from the USA and UK, as well as the dominance of US-based research in major paediatric surgical journals, leads to underappreciation of these relative incidence variations. Local research and opportunities for trainees to attend local conferences where this information is shared is essential to stimulate awareness of these issues.

### 2.2 Awareness of paediatric surgical conditions' management on the environment

Gastroschisis management in many settings has evolved in favour of sutureless non-operative management, with as many as two thirds of patients thus avoiding general anaesthetic as neonates, and only 10% of these needing surgery later for umbilical hernia repair (21). This technique for abdominal closure reduces ventilatory requirements as well as hospital costs (22). However, due to delayed arrival of our patients from off-site maternity units due to our paediatric hospital being free-standing with high demand for in-house intensive care or neonatal high care beds, and the majority of our patients requiring surgical extension of the defect to prevent bowel ischaemia prior to transfer, our unit has applied this approach to only a small number of patients. Although abdominal closure is a relatively minor procedure for uncomplicated gastroschisis, nevertheless the operating theatre exposure increases financial as well as environmental costs.

Seven to 30% of hospital waste comes from the operating theatre (23, 24). While our hospital has active recycling and renewable energy programmes, awareness of these and their impact is limited amongst staff including trainees. Our hospital uses reusable cotton surgical drapes and gowns primarily rather than more robust single-use adhesive plastic drapes. Reusable containers as well as medical grade paper are favoured rather than polypropylene blue wrap for sterilized equipment and linen, segregation of non-infectious waste is carried out and there is an onsite recycling programme for contaminated waste (reducing incineration smoke). However, many staff are unaware and dispose waste in the incorrect collection bins. Staff, trainers and trainees clearly ideally need education on relative carbon footprints (e.g., anaesthetic gases, laparoscopy running costs) and ecological hazards of theatre consumables as well as environmental and financial metrics of duration of intensive care, high care and general hospital stay to guide peri-operative decision-making.

Even with education, in the LMIC environment, certain processes are difficult to change such as the use of carcinogenic ethylene oxide gas sterilization to resterilise some “single use” plastics and other equipment, as other effective low-temperature sterilization techniques are prohibitively expensive. A controversial aspect of management differences between LMIC and HIC settings is the high use of disposable equipment in HIC, particularly in the sterile operative theatre environment. Device use in paediatric surgery remains low compared to adult surgery due to the limited size range of devices, especially for neonates where smaller items are required. The lack of these items contribute substantially to the surgical patient workload, yet, their use contributes to costs and medical waste. For this reason, re-sterilization of “single use” equipment is a common practice in LMIC settings, including for plastic disposables—but is being hindered by industry with the advent of “improved” devices electronically programmed to allow for a single use only, this to drive sales. In the example of gastroschisis, this also applies in some centres to the gastroschisis “silo bag”, a preformed silastic bag with a spring-loaded mouldable open base designed to slide over exposed bowel and aid visceral reduction in the condition, reducing the need for surgical abdominal closure. Some LMIC centres are unable to afford even resterilized bags, however, and fashion make-shift bowel coverings out of intravenous fluid bags or other plastic such as female condoms.

There is scope for increased reduction, reuse, more targeted recycling and revised environmentally sustainable practice in Paediatric Surgery. Sensitization of the medical undergraduate or paediatric surgical postgraduate trainee to cumulative waste/ resource utilization is important for sustainable healthcare and must be framed as more than just a financial decision. Currently, in LMIC countries, decision-making is driven chiefly by direct financial costs, and to a lesser extent, also influenced by convention, speed and convenience, and where known, clinical outcomes. Ecological impact consideration brings a new, as yet mostly unconsidered, paradigm. Where there appears to be conflict between these, there is rarely evidence in the LMIC to guide choices. A culture of solution-seeking in co-operation with industry in all economic situations can lead to more re-usable devices. Exposure of hidden ecological burdens of the healthcare system requires multidisciplinary engagement to determine the relative carbon footprint of various operating theatre costs, from anaesthetic gases to instrument sterilization and laundry, electricity, infectious and non-infectious waste disposal etc.

## 3 Knowledge

Measurement of the relationship between the climactic environment and the healthcare system is often hampered by interplay with socioeconomic factors, yet measurement and reporting of outcomes is one of the most effective educational tools in shaping sustainable developmental change (25).

### 3.1 Identifying gaps in knowledge

Inadequate educational exposure for all surgical team members has been identified as a critical barrier to implementing environmentally sustainable measures in the operating room, affecting over two thirds in a United Kingdom survey, which saw no sustainability measures implemented in 30% of surgeon responses (26). Lack of data to guide quantified decision-making is a major challenge to environmental stewardship. Critical thinking is required to measure the impact of current practices, identify areas of practice that may be harmful and amenable to change, and motivate for financial and other resource investments in stimulating change. This requires a foundation of core principles of surgical management through didactic training as well as understanding what is amenable to change through challenging trainers and students to analyse and appraise current practices.

In the context of gastroschisis, soft tissue infections are more common in hot “tropical” climates, with increased bacterial growth on skin in warm environments. Does this contribute to increased colonization of staff and parents and affect central line and wound infection rates even in the air-conditioned environment of a hospital? Limited data from a high income context (HIC) does show significantly increased gram-negative central line infections in hot weather (27). However, this is not something mentioned in paediatric surgery textbooks. LMIC academics must strive to bring these relevant local insights, and associated research into how to mitigate this, into the educational arena despite the clouding of interpretation of data coming from the overlap in geographic distribution of under-resourced LMIC and “tropical” climates. Other examples of knowledge gaps related to gastroschisis management in our setting include:

- Is it cheaper to buy breastmilk pumps for babies with gastroschisis to reduce the use of formula feeding and associated costly necrotizing enterocolitis and formula milk purchases?
- Do cheaper plastic central lines for parenteral nutrition in gastroschisis withstand alcohol locks to prevent sepsis and prolonged use in babies with short bowel syndrome adequately?
- Do these plastic lines have an impact on the patient compared to silicone lines?

### 3.2 Teaching critical thinking about environmental impacts

Apart from promoting a receptive attitude towards questioning in an education institution, critical thinking skills may need to be actively fostered to address the high novel information load brought by environmental considerations (3). Increased large language model machine learning tools afford rapid interrogation of scientific and grey literature only if the correct terms and understanding can be used to phrase questions appropriately (28). This requires didactic teaching of technical terms as a medical language as well as “big picture” thinking, which may need to be actively fostered through training in genesis of differential diagnoses using Socratic dialogue (29, 30) and “right brain” exercises (e.g., improvisation, visual thinking strategies) to promote observation and application skills (31, 32).

Mnemonics like “plastic is fantastic” can help a medical student remember that exposed hollow viscera prolapsing out of the congenital abdominal wall defect in gastroschisis is best protected this way (33) (see Figure 2), and may aid reduction of fatal hypothermia (34). Teaching this offers an opportunity to briefly acknowledge the less fantastic environmental impact of plastic (35), as well as consider biosafety and recycling potential of different types of plastic (36). Previously the need for sterility and medical-grade quality has been used as an excuse for ongoing *de novo* production of plastic and plastic-sheathed equipment, but students can be conscientized that science can be applied to create novel ways to reduce, reuse and recycle plastic and other materials (37).

**Figure 2:**
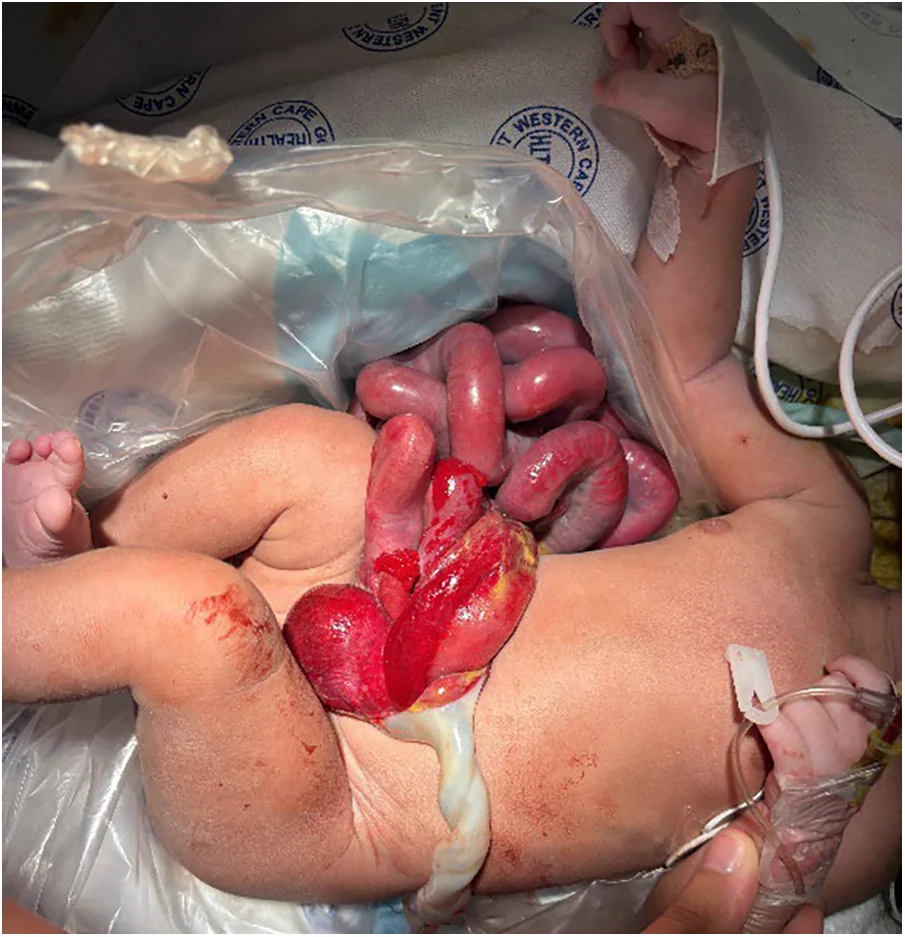
“Plastic is fantastic” to keep a baby with gastroschisis protected from evaporative fluid losses and dessication of exposed prolapsed hollow viscera…but different types of plastic have variable impacts on the infant and its environment. Picture used with parental permission.

## 4 Creating positive attitudes towards environmental sustainability

The ethical controversies highlighted above may make people uncomfortable about considering environmental footprints of diseases and their management. For example, choosing an expensive (plastic) disposable device vs. prolonging a surgical operation (26), particularly in LMIC settings where the costs of such devices are often magnified and staffing costs may be lower compared to HIC, poses a distributive justice dilemma. Similarly, jettisoning a piece of plastic that eases patient care may seem trivial compared to other healthcare concerns unless seen in the context of the greater environment (26). Creating positive attitudes towards environmental stewardship starts with education about the cost of ignoring it, as well as acknowledging value in exploring alternatives (3). This requires an educational environment that fosters questioning attitudes, multidisciplinary engagement, challenges top-down/ paternalistic attitudes, and individual appreciation (3). It also requires a strong sense of moral value-holding (38), for example to create a united front against a multi-billion-dollar industry in demanding increased reusability and recycling programmes for many of these devices as a new industry standard (39).

Experiential learning can positively shape attitudes towards effective environmentally friendly endeavours if they are framed appropriately (40), such as an enjoyable experience in the laparoscopic skills laboratory using a low-cost model made from recycled and locally available materials as shown in Figure 3 rather than a costly computer simulator.

**Figure 3:**
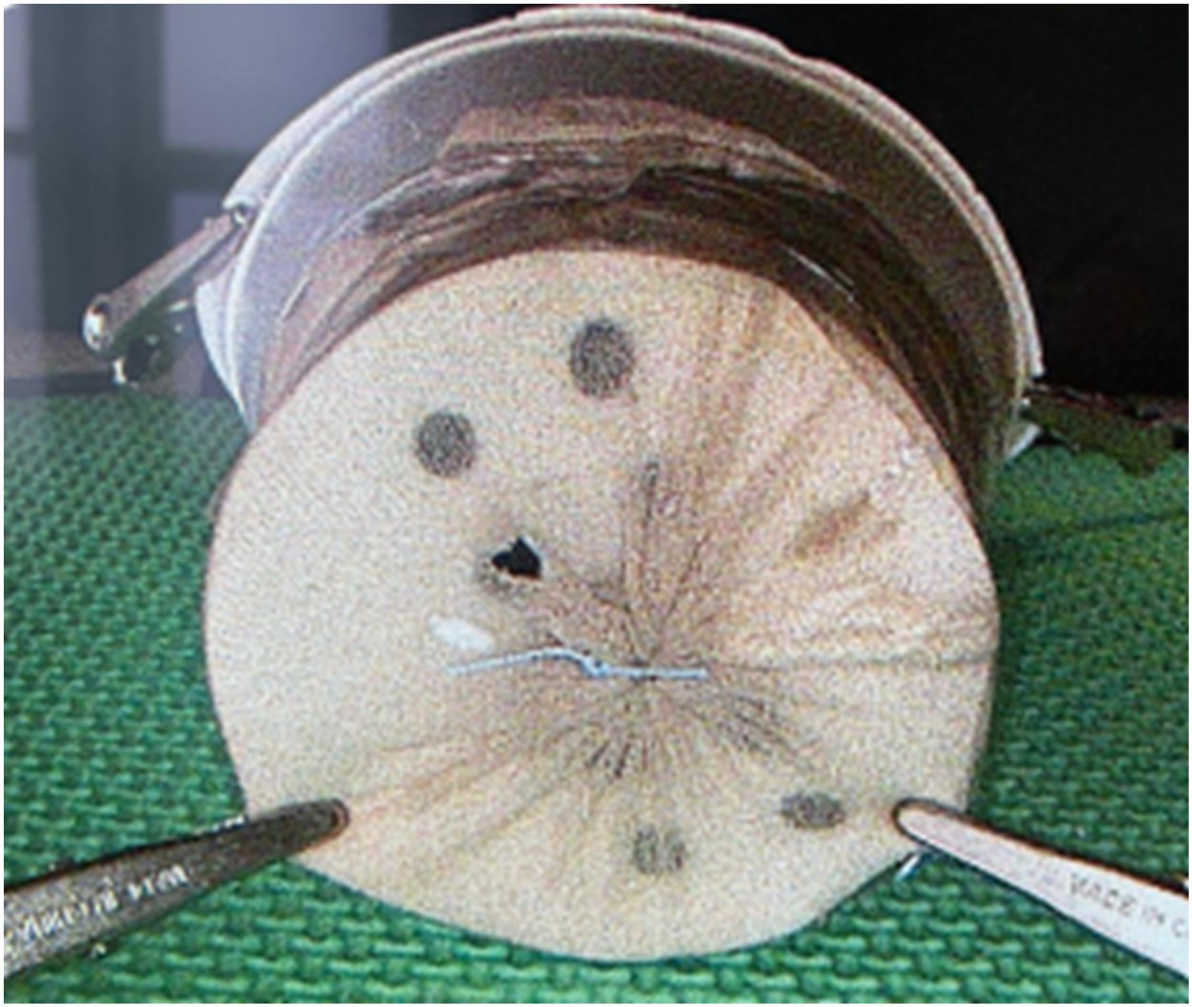
An effective low-cost laparoscopic skills training model using recycled polystyrene cups, cosmetic pads previously used for another skills application and nylon stocking can create positive associations with recycling.

### 4.1 Setting an example

Our hospital has reduced water usageby changing hospital faucets and using posters promoting increased alcohol hand rub for hand hygiene rather than washing. Additionally, major electricity savings have been achieved by installing solar panels our hospital car park (41), transitioning to light-emitting diode light fittings and placing timers onto wall-mounted instant boiling water dispensers. While these actions have positive environmental implications, without systematically informing staff and patients alike about both the financial and environmental benefits of these changes, individuals are left to come up with their own narratives on their motives and benefits.

Our unit has abandoned paper materials for undergraduate medical students, including in-house textbook material, logbooks and attendance registers, in favour of digital versions. However, unless digitalization is mainstream across the medical curriculum, the carbon footprint of the digital devices and electricity required to access these is not mitigated (42).

A major initiative towards bringing environmental considerations formally into the surgical spotlight systematically, which also requires concerted effort across all surgical disciplines, is a move to introduce a “Green” surgical checklist at the start of each operation (43). This is an addition to the current World Health Organization pre-operative checklist already used before each operative case in 70% of theatres around the world (44), but should be tailored to the local situation. Suggested content includes anaesthetic considerations, surgical preparation, intra-operative decision-making about consumables (including noting items that should not be opened until actually needed in case of changes in requirements), and post-operative waste management considerations as well as reminders to switch off screens and theatre lights once not in use (43). Application of the UK Royal Colleges of Surgery Green Preoperative Checklist has shown mixed success, with potential for a 50% of further reduction in carbon emissions identified if full compliance can be achieved, notably around single-use laparoscopic equipment and drapes as well as unnecessary antibiotic use and opened unused equipment (45).

### 4.2 Educational initiatives

Concerted educational initiatives are required at all levels of healthcare workplaces from direct educational communication for faculty development as well as providing resources such as online learning programmes, newsletters and posters to highlight both environmental challenges and actionable steps (46). Publicization of successes is an opportunity for community engagement and can enhance community satisfaction as well as potentiate multi-sector engagement in spending of both financial and time resources towards furthering various environmental sustainability goals (47). These indirectly benefit trainees at the healthcare institution as part of the healthcare community, supporting direct changes from curricular changes and institutional policy changes which require regulatory body action (46).

Sustained reflective physical behaviour from washing hands after a patient interaction to correctly disposing recyclable waste material in colour-coded bins benefits from a multimodal approach rather than just visual reminders such as posters (48). Environmental discourse thus needs to be integrated on all levels of the curriculum, including the cognitive, affective and psycho-motor domains of Bloom's educational taxonomy, to be effective (49). This can be done through integrating environmentally friendly practices into other practical training, such as discussions regarding the advantages and disadvantages of using reusable vs. plastic disposable sterile equipment during training on gastroschisis silo application or central line insertion skills, as well as throughout supervised day-to-day clinical practical patient care.

## 5 Skills to manage environmental sustainability in paediatric surgery practice

### 5.1 Teaching research skills to measure impact

This should be a major responsibility of academic institutions. Increasingly obligatory Masters theses for South African surgical trainees are looking at topics such as carbon footprints of various hospital practices (50), epidemiology and patient-centred outcomes within disciplines. This may require multidisciplinary collaboration.

Looking at gastroschisis again, the “Bogota Bag” (51) and its modifications are innovative life-saving low-cost solutions for temporary abdominal closure, using an intravenous fluid bag usually sutured to the abdominal sheath to cover prolapsed viscera, in babies with gastroschisis where a preformed silicone silo bag is unavailable or inappropriate (52, 53). However, phthalates from the bag's PVC content are absorbed to an unknown extent by the exposed viscera (54). These “forever chemicals” may hypothetically carry a risk of organ dysfunction, infertility and carcinogenesis later in life (35). It is unknown if this consideration is important in the context of a young child where the high metabolic rate and relatively large surface area as well as early age of exposure may magnify effects compared to adults undergoing the same procedure. Clinical studies on long-term impacts are unlikely to be feasible or practical. Scientific advances on acute measurement of plastic absorption may aid future decision-making within these resource-constrained environments where commercial abdominal closure products (made from silicone or safer plastics) are not available or affordable, e.g., whether lining the cut IV bag used in make-shift management of the open abdomen such as in gastroschisis with a safer polyethylene adhesive clear film dressing reduces this absorption. Discussions of this nature require practical, multidisciplinary approaches as well as funding and dedicated protected research time during training to enable actionable outcomes.

### 5.2 Teaching personal responsibility

Even without embedded environmental considerations within the curriculum, individual paediatric surgeons can promote environmental awareness, which can filter through to all levels as a culture of environmentally considerate practice. This can be motivated through appealing to personal professionalism, personal as well as institutional obligation, and leadership responsibilities amongst others (55, 56).

Personal responsibility and self-proclaimed knowledge are key to changing plastic consumption and waste management on a personal level (57). Growing a sense of personal ownership for resource management may require modelling, from seniors especially, through environmentally sensitive dialogue and discussions, as well as willingness to share and highlight personal lifestyle choices. Sensitization from small personal initiative such as “bring your own coffee cup” to admonishments to “switch off the lights when you leave the lecture hall please” can help build narratives of personal agency, as well as community cultural acceptance, which can grow to narratives of institutional responsibility and agency.

### 5.3 Teaching advocacy skills

Personal agency does not necessarily translate to individuals tackling sustainable practices on an institutional level, for which a multifaceted approach is required including development of advocacy skills (58) and effective knowledge sharing to build cultural acceptance of “green” initiatives. In minimally invasive surgery, operative time may be significantly expedited by single-use energy devices, stapling devices and other expensive equipment. These devices result in a high burden of plastic waste, magnified by the sterile packaging required. While the convenience and safety of single-use may seem cost-efficient due to reduced operative time-costs, the lucrative nature of single-use handpieces has mainly deterred the development of reusable devices. Advocacy skills and interdisciplinary and cross-tier healthcare system communication skills are required to pressurize industries to partner for more sustainable solutions.

Communication through presentations, interpersonal lobbying and interdisciplinary collaboration can all be enhanced through active training. This may require upskilling of educators as well as allocating time within training programmes for such “soft” skills. Seniors rarely discuss mundane administrative tasks with juniors but task-shifting some of these to training doctors under supervision can help to teach skills ranging from the format of a motivational letter to how to generate specifications for procurement of an environmentally friendly reusable device, as well as presentation of scientific data in a succinct and audio visually appealing manner. Advocacy training can include informal comments during presentation of the regular curricular programme, formal lectures and workshops, positive valuation of advocacy role models through newsletter features and special invited lectures etc.

While resterilization of single-use medical devices is practiced in LMIC settings out of frugality, many regulators including the South African Health Product Regulatory Authority regard this as malpractice (59). There is however recognition by the United States of America Federal Device Authority acknowledging that it is usually low risk (60). Organized advocacy to review and promote safe reuse and recycling of devices requires interplay between the end-users, governmental and industry regulators and the medical device industry. Safe and effective reutilization needs to be driven by environmentally-concerned end-users (59) as other stakeholders are not currently incentivized to do so.

## 6 Participation: rewarding and promoting behaviours

### 6.1 In-house

Sustained exposure to a subculture that regularly acknowledges the impact of adverse interplays between the environment and healthcare workers, the healthcare system and patient and disease factors (“green consciousness”)is associated with increased action-taking to mitigate them (61). Praising a trainee who has successfully inserted a peripheral-inserted central catheter for a baby with gastroschisis needing parenteral nutrition might include highlighting that the resultant avoidance of a general anaesthetic for a more invasive line also reduces greenhouse emissions (62).

Critical thinking about environmental sustainability requires positive training and work environments that embrace questioning. This requires knowledgeable and appropriate-level educators, who are willing to promote understanding and consideration of environmental-healthcare system interplay within the existing curriculum even where data is still limited to scientifically guide choices (3). Compassionate, rational decision-making must be demonstrated as ethical dilemmas are exposed throughout the complex field of Paediatric Surgery. For example, should a gastroschisis survivor who has had a prolonged and complex postnatal hospital stay travel 700 km back to a tertiary hospital for follow-up care or is local follow-up with a rural clinic nurse acceptable? Didactic teaching is not enough to teach these skills; real world case studies should be encouraged to integrate these concepts for trainees to navigate these decisions (3). Patient interaction is also required on an undergraduate level to appreciate social impact on disease and build empathy, which is essential to effective implementation of systemic environmental reforms (3).

Senior clinician and hospital and university management modelling of ethical and sustainability-minded behaviour is essential to build an ethos of conscientious surgical resource use (25). In the LMIC environment with inadequate skilled human resources presenting great barriers to care, a high level of personal integrity must be emphasized and acknowledged to reduce opportunistic shortcuts. A culture of openness and respectful engagement at all levels is also needed, to create an environment where old practices can be carefully examined, potential harms acknowledged and new alternatives sought despite the potential disruption this creates. Diverse participation such as use of medical students to assist in research projects can increase fruitful innovation, as can use of low-cost technology such as publicly available artificial intelligence tools in research and audits to create much needed evidence for best clinical and environmentally sustainable practice despite limited research infrastructure.

### 6.2 External

Community education on environmental care imperatives foster institutional accountability and transparency which increase the viability of sustainable development initiatives (63). Public health management requires stepping out of single discipline silos for best care. While there is growing awareness of the long-term impact of high use of toxic phthalates and polyvinyl carbons in the medical industry on individual health (35, 54), surgeons in our setting are not conscientized or educated about these. Cross-disciplinary ward rounds can create awareness of new environmental concerns. Invited guest lecturers with social or environmental advocacy bents and accolades should be sought out from other disciplines. Putting these into an already full curriculum is challenging but places value on interdisciplinary engagement. Hospital- and university-level awards and publicity, which may not necessarily have monetary value associated in an LMIC environment unless through community donations, can nevertheless create community awareness and build empathy and support towards “green warriors” who stand out in championing ecologically sound practices and related education and/or research.

Time limitations and potential conflicts of interest for busy educator-clinicians may limit capacity for direct engagement of medical industry role-players, but liaison within the government as well as non-governmental sector could help to bridge this necessary gap and facilitate dialogue towards more innovative, cost-effective ways to reduce particularly plastic waste and other procedure-related environmental contaminants.

Some of our current and potential paediatric surgical curriculum educational learning points and opportunities related to environmental sustainability are summarized in Table 1. These may not be globally applicable.

**Table 1:** UNESCO education pillars for sustainable development (EPSD) for existing (●) and potential (o) environmental sustainability learning opportunities in paediatric surgical curricula, with pedagogical techniques highlighted in bold.

Unesco epsd	Awareness	Knowledge	Attitude	Skills	Participation
Under-graduate medical student training	Teratogens can cause birth defects Online pedagogical resources are available	Clinical diagnosis with few special investigations for most paediatric surgical conditions	Own narratives about water and electricity saving (e.g., budget driven)	Basic recycling Self-advocacy	Switch off lights not in use Bring own bottle or mug Digital online study resources Utilize 2nd-hand scrubs and medical equipment Lift clubs
	○ LECTURES & TEXTS highlight environmental teratogens *(e.g., maternal petroleum product exposure)* ○ INTRODUCTORY DOCUMENTS highlight CO_2_ footprint reduction of ↑ volume digital book use	○ LECTURES, TEXTS, TUTORIALS: Surgery is costly & ↑ CO_2_ footprint but no/ poor/ late surgical care ↑↑ costs *(e.g., delayed diagnosis biliary atresia leads to liver transplant)*	○ POSTERS in educational & healthcare institutions: educators value & promote care for environment ○ EDUCATOR ATTITUDE modelling positive approach	○ EDUCATOR ATTITUDE advocate for patient & environment ○ PATIENT CASE STUDY includes social & environ-mental enquiry: ↑ advocacy & awareness	○ POSTERS: Colour-segregated waste-bin separation in healthcare environment ○ PATIENT CASE REPORTS: consider environmental & social impact of disease and vice versa
Post-graduate specialist paediatric surgeon training	Weather and socioeconomics affect patient presentation Operating temperature impacts outcomes	Organized & green environment enhances wellbeing & safety	Special devices & disposable plastic cost money but save time	Budget-based decision-making Correct waste segregation Conscientious disposable consumable use	Avoid plastic lids for take-away coffee; use own cup Communication about environmental health risks to self
	○ DIDACTIC & BEDSIDE TEACHING: Epigenetic causes and effects on surgical disease; plastic ○ JOURNAL CLUB REVIEW: CO_2_ surgery footprint	○ POSTERS/ CIRCULARS: theatre running & consumable cost awareness	○ FORMAL LECTURES: infection control, environmental impacts of resource utilization embedded in curriculum	○ Estimate relative environmental impact of procedure components ○ Discuss environmental aspects of care with patients & undergraduates	○ ETHICAL DIALOGUE: participate in multidisciplinary discussions on ethical dilemmas on resource stewardship ○ GREEN COMMITTEES: create sustainability initiatives, quality improvement projects
Training of trainers (qualified paediatric surgeons in academic practice)	Teratogens & socioeconomic impact on LMIC outcomes	Disposable equipment is expensive Reusable drapes and surgical equipment are available	Leadership & critical thinking skills Ethical approach to safety and long-term outcomes for individuals	Waste segregation ↓ unnecessary procedures& consumables Communication about risks	Efficient instrument use Budget-conscious approach reduces waste and consumable use
	○ INTERDISCIPLINARY INVITED LECTURES, WORKSHOPS, INFORMAL MEETINGS ○ RESEARCH: local CO_2_ footprints, teratogens, LMIC patterns & environmental correlates	○ POSTERS/ REFERENCE DOCUMENTS: itemize consumables & theatre running costs, CO_2_ burdens ○ RESEARCH: INFORMAL MULTIDISCIPLINARY ENQUIRY: waste management	○ MULTIDISCIPLINARY COLLABORATION in ethical and sustainability discussions ○ GREEN OPERATIVE CHECKLIST leadership through implementation and diligent participation	○ RESEARCH/ QUALITY IMPROVEMENT: Audit resource use/ CO_2_/ waste ○ SOCRATIC DIALOGUE: discuss sustainability with staff & students; build awareness, respect & sense of professional responsibility	○ NEGOTIATE WITH INDUSTRY & GOVERNMENT *to regulate device recycling* ○ PRESENT RESEARCH: local CO_2_ footprints, teratogens, LMIC patterns ○ CHARITY DRIVE POSTERS: Donate lightly used scrubs & medical equipment ○ INSTITUTIONAL POLICY & CULTURE: hand hygiene instead of plastic gloves for dry unsterile patient contact

## 7 Conclusion: actionable recommendations

Table 1 summarizes training opportunities to integrate environmental education into Paediatric Surgery. The existing curriculum of Paediatric Surgery, as demonstrated by gastroschisis as a highly relevant study topic, is sufficient to foster interrogation of all aspects of sustainability of healthcare and the environment, but this requires generating a sense of personal agency and responsibility towards environmental management at all levels of healthcare and healthcare training. Opportunities to expand Paediatric Surgical teaching on environmental sustainability, and healthcare-environmental interplay, require willingness to embrace change, interaction with other disciplines and a high level of critical thinking. Most importantly, a culture within the healthcare sector of valuation and promotion of environmentally sustainable practice, led by senior clinicians and hospital and university management, must underpin the Paediatric Surgical educational setting, as many aspects of both undergraduate and postgraduate training are implicit modelled attitudes or opportunistic, contextualized in bedside or operating room teaching. Measurement of the impacts of daily decision-making in paediatric surgery through LMIC-focussed research on practical concerns requires academic, financial and other practical support, and is needed to improve individual patient and broader environmental outcomes.

As changes and uncertainties about the future across all levels of the environment grow, training paediatric surgeons also need to learn how to promote agency, adaptation and resilience in patients and their families as well as themselves.

## 8 Discussion limitations

Formal study of environmental impacts in paediatric surgery, and education on this within the surgical milieu, is limited. Specific data on current and potential impact of care for paediatric surgery in our setting is lacking. This article extrapolates data from high income countries and other arenas of healthcare and education related to environmental sustainability. There is no government-level or university-level agreement on overarching principles for weighing the environmental merits and detrimental impact of various healthcare practices, particularly in paediatric surgery. Similarly, data on effectiveness of various pedagogical techniques to teach the EPSD are lacking. Locally relevant, specific data will require funding input, ethical engagement and industry, government, academic and non-governmental organization collaboration to overcome industry-related adverse incentives affecting all levels of paediatric surgical care, within the greater framework of healthcare.
